# Distinguishing chronic myeloid leukemia in megakaryocytic blast crisis from *de novo* Ph+ acute megakaryoblastic leukemia: a case report and systematic review

**DOI:** 10.3389/fimmu.2026.1812840

**Published:** 2026-07-29

**Authors:** Qingqing Liu, Pu Yu, Xiaozhen Li, Hai Lan, Zenghui Liu

**Affiliations:** 1 The First Clinical Medical School of Guangzhou University of Chinese Medicine, Guangzhou, China; 2 Department of Hematology, the First Affiliated Hospital of Guangzhou University of Chinese Medicine, Guangzhou, China; 3 Department of Hematology, the Shunde Hospital of Guangzhou University of Chinese Medicine, Guangzhou, China

**Keywords:** acute myeloid leukemia, chronic myeloid leukemia, differential diagnosis, megakaryocytic blast crisis, Philadelphia chromosome, tyrosine kinase inhibitor

## Abstract

Chronic myeloid leukemia (CML) with megakaryoblastic blast crisis (MKBC) as the initial manifestation is extremely rare, accounting for less than 3% of all CML cases. Philadelphia chromosome-positive acute myeloid leukemia, FAB M7 subtype (Ph+ AML-M7), is also known as Philadelphia chromosome-positive acute megakaryoblastic leukemia (Ph+ AMKL), representing a distinct and prognostically unfavorable category of AML. Morphologically and immunophenotypically, these two entities are nearly identical, posing significant diagnostic challenges. We describe a novel case of Ph+ leukemia with MKBC differentiation that appears most consistent with CML in blast phase (BP). Following treatment with a tyrosine kinase inhibitor (TKI) combined with induction and consolidation chemotherapy, the patient achieved complete remission (CR). Although hematopoietic stem cell transplantation (HSCT) was declined due to economic constraints, the patient has maintained deep molecular remission(MR5, BCR::ABL1^IS^ ≤ 0.001%)for 35 months to date. Through a systematic review of existing literature, this article elucidates key discriminative features between the two conditions and proposes a practical diagnostic and therapeutic framework to guide clinical decision-making.

## Introduction

Chronic myeloid leukemia (CML) is a myeloproliferative neoplasm driven by the BCR::ABL1 fusion gene, typically progressing from chronic phase (CP) through accelerated phase (AP) to blast phase (BP) if untreated (1, 2). BP is defined by the presence of ≥20% blasts in peripheral blood or bone marrow (BM) and is associated with poor prognosis. Among various differentiation lineages, megakaryoblastic blast crisis (MKBC) is exceedingly rare, accounting for less than 3% of all CML transformations, and cases presenting with MKBC as the initial manifestation are even rarer (3). Philadelphia chromosome-positive acute megakaryoblastic leukemia (Ph+ AMKL) is a distinct entity that also presents with megakaryoblastic differentiation and carries the t (9;22) translocation but lacks a prior CP history of CML.

Distinguishing CML-MKBC from *de novo* Ph+ AMKL is clinically challenging yet critical, as treatment strategies differ substantially. Herein, we report a rare case of Ph+ leukemia with MKBC differentiation that appears most consistent with CML-BP. We systematically review the literature, compare clinical, immunophenotypic, and molecular features between these two entities, and propose a practical diagnostic and therapeutic framework to assist clinicians in this challenging differential diagnosis.

## Case presentation

**Figure 1:**
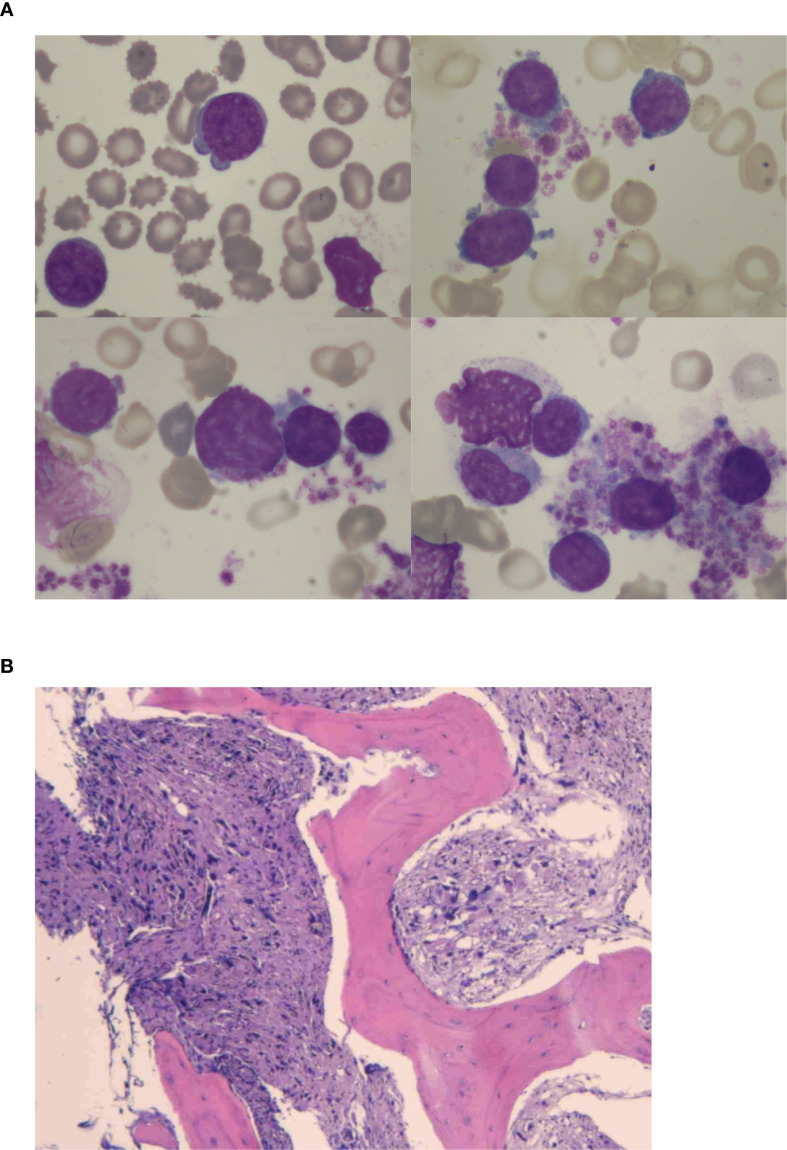
**(A)** Bone marrow aspirate smear (Wright-Giemsa stain, ×1000) showing leukemic blast cells and megakaryocytes. These blasts exhibited rounc nuclei with fine chromatin and basophilic cytoplasm, some showing cytoplasmic blebbing. **(B)** Bone marrow biopsy (HE stain, ×200) showing diffuse fibrous tissue hyperplasia and scattered atypical nucleated cells; inset: reticulin stain (+) indicating grade III fibrosis.

**Figure 2:**
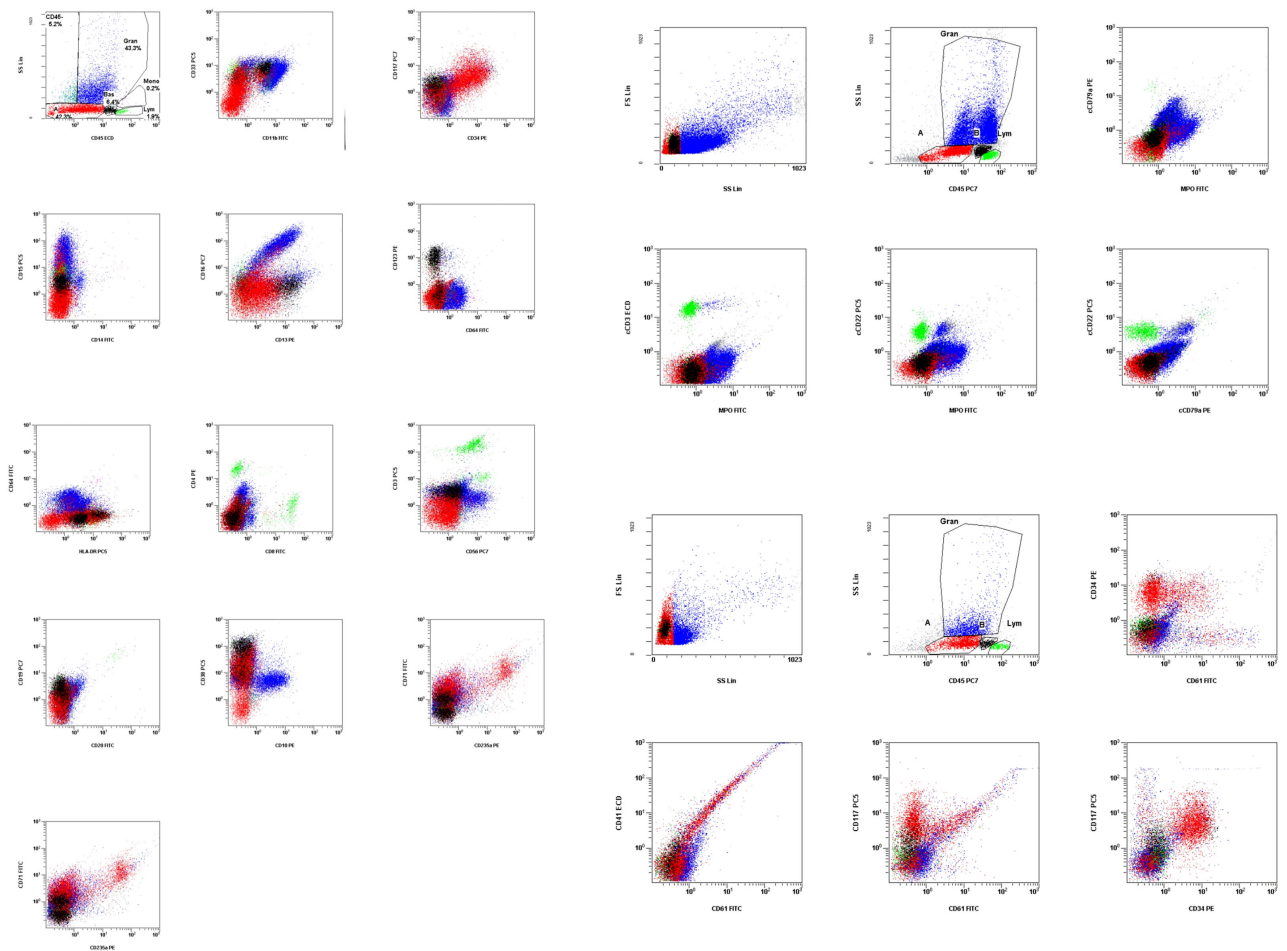
Flow cytometry was performed on bone marrow aspirate using a CD45/SSC gating strategy, a distinct blast population (Population A) was identified in the CD45-dim/SSC-low region, accounting for 46.3% of total nucleated cells, immunophenotyping analysis of the blast gate showed negativity for CD56, CD20, and CD79a, and positivity for HLA-DR, CD34, CD117, CD13, CD33, CD41, CD61, and CD38.

**Figure 3:**
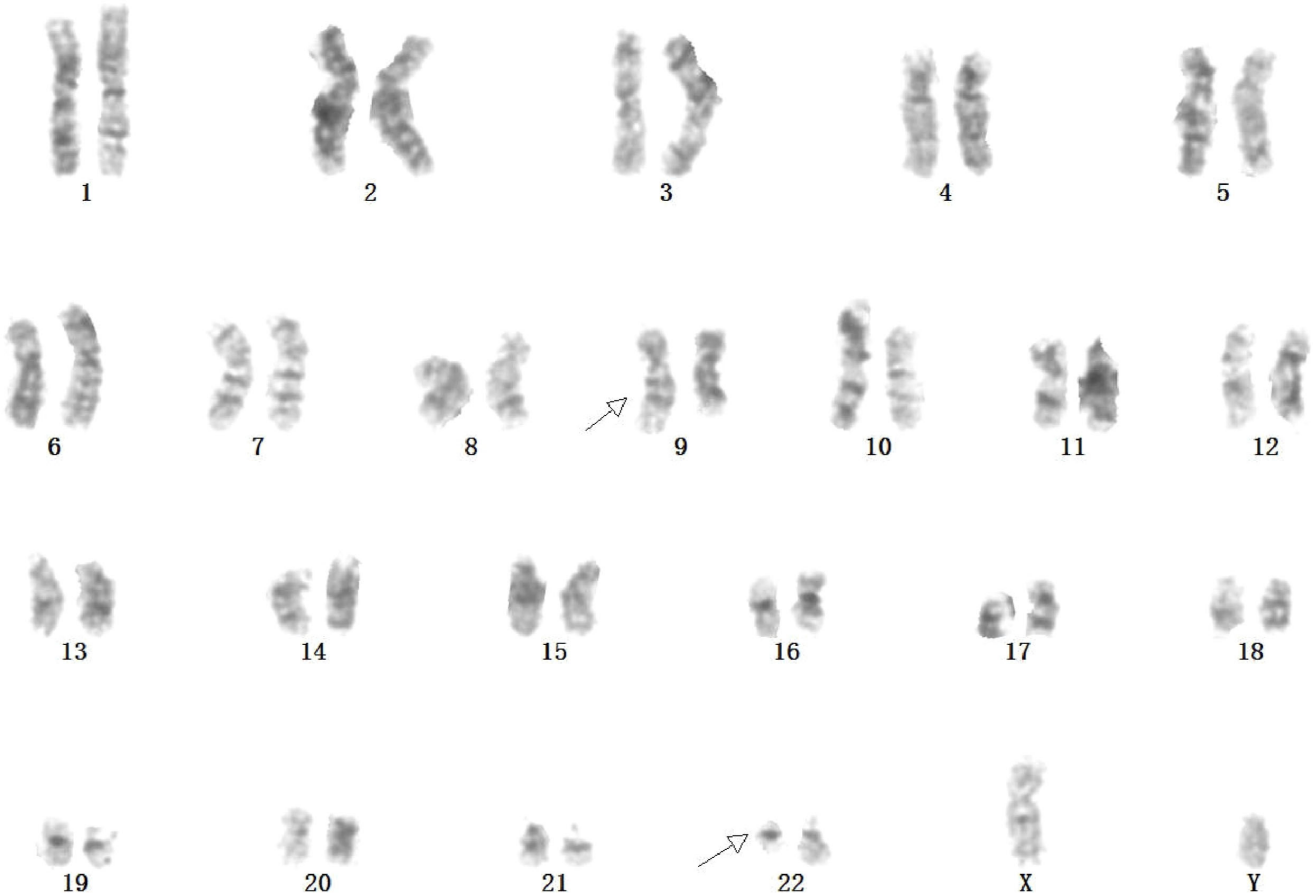
Chromosome karyotype 46, XY, t (9; 22) (q34; q11.2) [15]/92, XXYY, t (9; 22) (q34; q11.2) x 2 [2]. 46, XY, t(9;22)(q34;q11.2) in 15 of 17 metaphases, the remaining two metaphases showed a tetraploid karyotype 92, XXYY, t(9;22)×2, and translocation between 9q34 and 22q11.2.

A 49-year-old male presented to the Department of Hematology, first Affiliated Hospital of Guangzhou University of Chinese Medicine, in February 13, 2023, reporting a one-month history of upper abdominal distension and pain. The patient had no record of hematological abnormalities before admission, and there were no antecedent features of CML-CP (e.g., fatigue or night sweats) reported by the patient. Laboratory investigations revealed leukocytosis (white blood cell count 58.63×10⁹/L) and anemia (hemoglobin 74 g/L). Abdominal ultrasound demonstrated marked splenomegaly. Peripheral blood smear revealed 22% blasts with megakaryocytic features and 8% basophils. BM aspirate smears showed marked megakaryocyte proliferation with dysplasia. Megakaryoblasts accounted for 52% of nucleated cells, basophils were markedly increased, constituting 10.5% of nucleated cells (**Figure 1A**). BM biopsy demonstrated grade III myelofibrosis, CD61+ atypical cells, and markedly reduced granulocytic (MPO+) and erythroid (CD71+) series by immunohistochemistry (**Figure 1B**). Flow cytometry immunophenotyping of the blast population revealed co-expression of megakaryocytic markers (CD41, CD61) and progenitor markers (CD34, CD117) with aberrant myeloid-associated markers (CD13, CD33). Myeloperoxidase (MPO) was negative, and B-cell markers were absent, effectively excluding alternative lineages (**Figure 2**). Immunohistochemistry confirmed the megakaryoblastic lineage of the infiltrating cells through CD61 staining at the tissue level and demonstrated severe suppression of normal granulocytic and erythroid hematopoiesis. Cytogenetic analysis showing a complex karyotype, including a hyper-tetraploid, double-Ph clone and translocation between 9q34 and 22q11.2, and no other cytogenetic abnormalities were found (**Figure 3**). BCR-ABL fusion gene screening was performed on BM aspirate using a multiplex real-time quantitative PCR panel for 56 leukemia-associated fusion genes. Diagnostic quantitative PCR detected BCR::ABL1(p210) transcript with a BCR::ABL1^IS^ of 144.21%. Next generation sequencing was performed using the hematopoietic and lymphoid neoplasms gene mutation panel (222 genes): ASXL1 NM_015338.6:c.1860_1861delTG (p.Ala621fs) (30.60%), ASXL1 NM_015338.6:c.1934dupG (p.Gly646fs) (12.64%), CSF1R NM_005211.3:c.2056_2079del (p.Ser686_Pro693del) (42.80%), PTPN11 NM_002827.4:c.167G>A (p.Arg56Gln) (8.70%), and RELN NM_005045.4:c.10108A>G (p.Thr3370Ala) (49.30%), and no other gene mutations were detected.

Although the patient’s BM morphology, flow cytometry immunophenotyping, absence of prior CML history, and BCR::ABL1(p210) positivity closely aligned with diagnostic criteria for Ph⁺ AMKL, the presence of splenomegaly and significant basophilia at presentation strongly supported a diagnosis of CML presenting with megakaryocytic blast crisis (MKBC) as the initial manifestation. Targeted fluorescence *in situ* hybridization (FISH) to determine whether BCR::ABL1 signals are present across differentiated myeloid lineages—considered a gold standard for distinguishing CML-BP from *de novo* Ph+ AMKL—was not available in our laboratory. Therefore, the diagnosis relies on a composite of clinical, morphologic, karyotype, and molecular features.

## Therapy and prognosis

**Figure 4:**
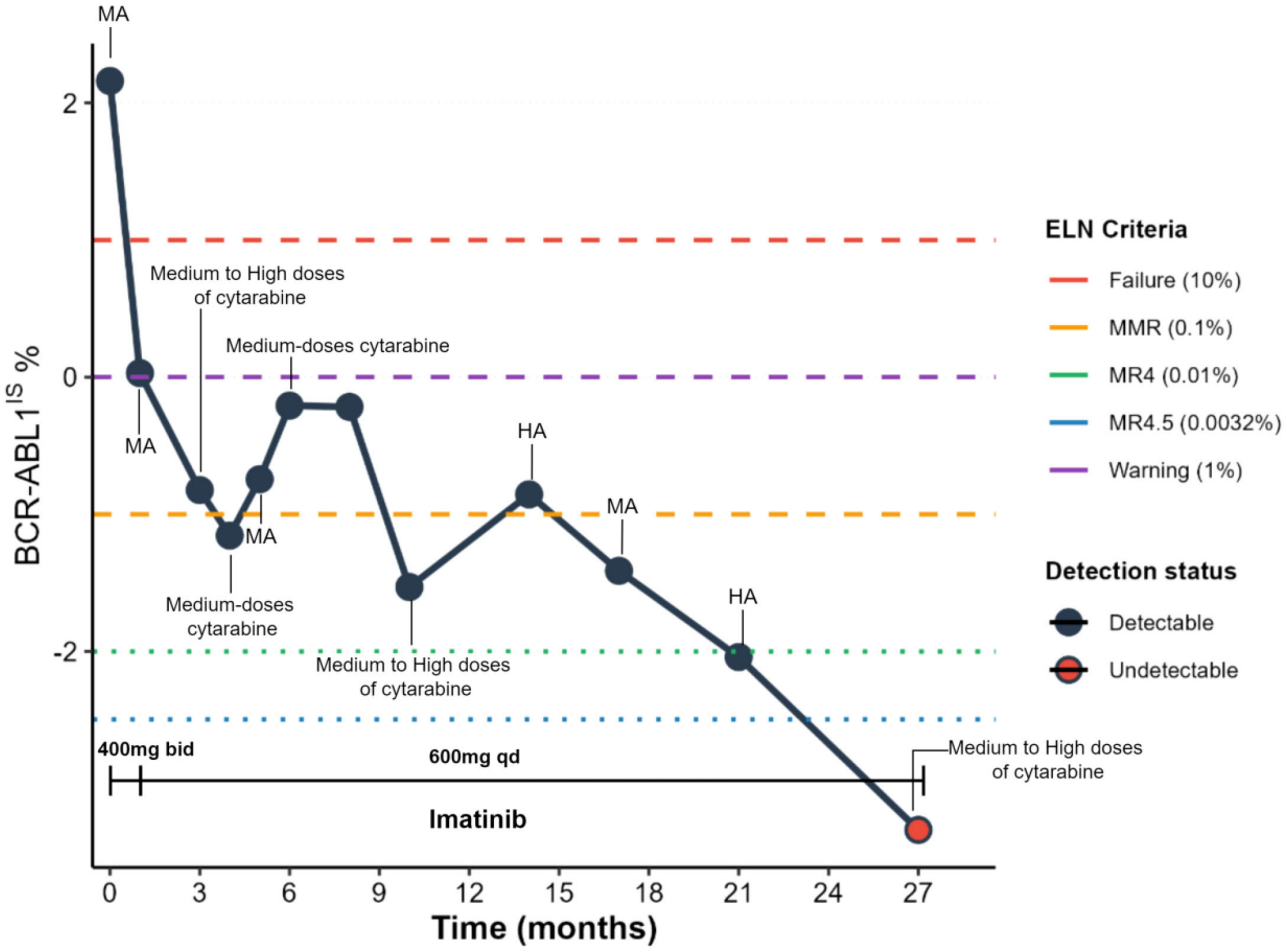
BCR::ABL1^IS^ values over treatment time.

The patient initially received induction chemotherapy with the MA regimen, consisting of mitoxantrone 10 mg/m² on days 1–3, combined with cytarabine 100 mg every 12 hours on days 1–7. Additionally, the tyrosine kinase inhibitor (TKI) imatinib was concurrently administered orally at a dose of 400 mg twice daily. One month later, the BM morphology confirmed complete remission (CR), and BCR::ABL1^IS^(p210) levels declined from 144.21% to 1.07%. He subsequently received alternating cycles of induction and consolidation therapy, with regular monitoring of BCR::ABL1^IS^(p210) (**Figure 4**). Between May and June 2023, consolidation therapy consisted of cytarabine (3.5 g IV every 12 h on days 1, 3, and  5) along with imatinib (600 mg once daily). Repeat BM examination demonstrated sustained CR, with BCR::ABL1^IS^(p210) further decreasing to 0.07%, achieving a major molecular response (MMR).

Given the high-risk profile conferred by adverse genetic abnormalities and the patient’s sustained CR status, hematopoietic stem cell transplantation (HSCT) was recommended. However, the patient declined due to financial constraints. Subsequently, from July to December` 2023, he received alternating cycles of the MA regimen and cytarabine-based regimens. Although hematologic remission persisted, subsequent monitoring revealed molecular relapse with BCR::ABL1^IS^(p210) rising to 0.18% and 0.62% over the following two months; therefore, from April to November 2024, the patient received two courses of the HA regimen (homoharringtonine 3.6 mg IV on days 2–6 plus azacitidine 100 mg subcutaneously on days 1–7) and one additional course of the MA regimen, while continuing imatinib (600 mg once daily).

During treatment, the patient developed grade IV myelosuppression complicated by Klebsiella pneumoniae septicemia, which resolved with anti-infective therapy and supportive measures (e.g., antibiotics and platelet transfusions). Flow cytometry in November 2024 showed undetectable blasts, and by May 2025, BCR::ABL1^IS^(p210) was undetectable. To date, the patient has maintained deep MR5 for 35 months according to the 2025 ELN recommendations for the management of CML (4).

## Discussion

### Diagnosis and differential

**Figure 5:**
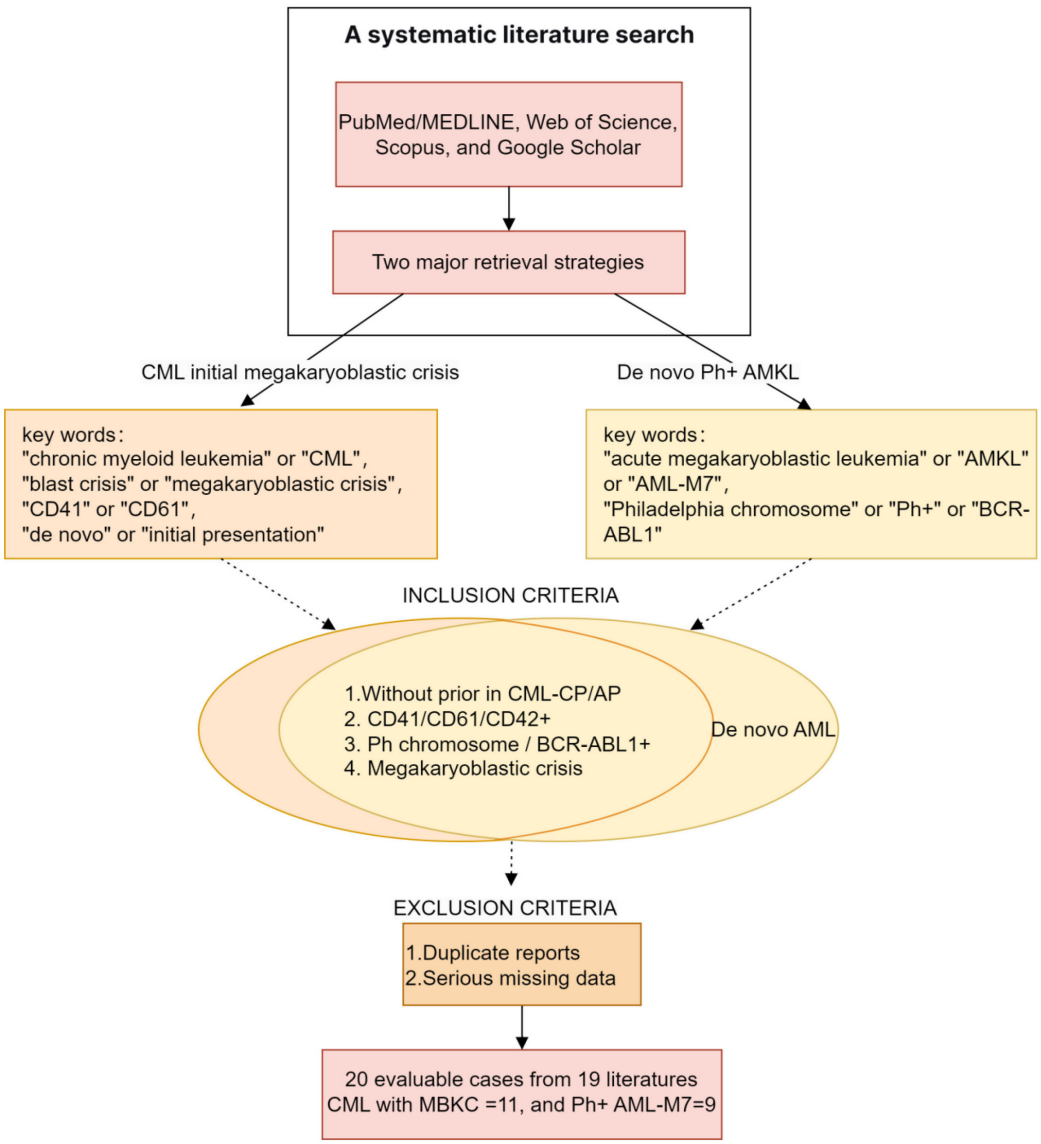
Decision tree for WHO/ICC classification of suspected CML with megakaryocytic crisis.

Ph+ AML was originally recognized as CML-BP but was recognized as a distinct entity in the 2016 WHO classification of Myeloid Neoplasms and Acute Leukemia (5), and there is no unified standard to distinguish CML-MKBC from Ph+ AMKL at present. In order to summarize the main distinguishing points of the two entities, a systematic literature search was conducted following PRISMA guidelines in PubMed/MEDLINE, Web of Science, Scopus, and Google Scholar from inception to February 2026 (**Figure 5**). A total of 195 records were identified, and ultimately 11 cases of CML with MBKC as the first episode (3, 6–15) and 9 cases of Ph+ AMKL have been reported (16–23). Data in the currently summarized literature are partially incomplete, we have collected recorded information (**Table 1**).

**Table 1:** Summary of CML with MKBC as the initial manifest and *de novo* Ph+ AMKL cases.

A. 11 cases of CML with MKBC as the initial manifest.											
Authors	Year	Peripheral		Splenomegaly	BM Characteristics					Treatment	Survival (moa^a^)
		WBCs (x10^9/L)	Basophils (%)		Blasts (%)	Immunophenotype	Cytogenetics	BCR /ABL	Fibrosis		
CD Wu et al. (6)	1996	19	2	No	dry tap	CD43, Factor VIII	46,XX,t(9;22)(q34.1;q11.2)	NA	ND^b^	Ara-C^c^+etoposide with HSCT	Died
Hirose Yet al. (7)	2002	2.6	1	Yes	20	CD13,CD33,CD34,CD41a,HLA-DR	NA	210	ND	VP^d^+MTX^e^+6- MP^f^IME^g^	1
Pelloso F et al. (8)	2002	11.4	8	Yes	23	CD13, CD33, CD34, CD41, CD45, CD61, HLA-DR	46,XX,t(9;22)(q34.1;q11.2)	210	2	DA^h^with IM	6
Campiotti L et al. (9)	2007	9.9	7	Yes	56	CD13, CD33, CD34, CD41, CD45, CD61, HLA-DR	46,XX,t(9;22)(q34;q11)	210	ND	IA^i^with IM	4
Bryant BJ et al. (10)	2007	23.8	11	Yes	dry tap	CD31, CD43, CD45	46,XX,t(9;22)(q34.1;q11.2)	NA	ND	Hydroxyurea and radiation	Died
Pullarkat ST et al. (11)	2008	16.2	0	No	dry tap	CD7, CD33, CD41, CD45	64,XY with duplicate Ph chromosome	NA	ND	IM	Died
Al-Shehri A et al. (12)	2010	36	4	Yes	50	CD41,CD42,CD61	46,XX,t(9;22)(q34;q11)	210	ND	ITA^j^ with IM+CBT^k^	22
Lu jin et al. (13)	2011	38.04	8	Yes	20	CD7, CD9, C13, CD33, CD34, CD38, CD41, CD61, CD117, CD123, HLA-DR	46XY,t(9;22)(q34;q11)	210	3~4	HA^l^DA with IM, Nilotinib	NA
Karkuzhali P et al. (14)	2013	27.9	4	Yes	dry tap	NA	NA	210	2	ADE^m^ with IM	Died
Sasaki H et al. (15)	2019	52.4	29	Yes	30	CD33, CD34, CD42b, Factor VIII, MPO	NA	210	2	Dasatinib,Ponatini b,HSCT,CBT	12
Agrawa S et al. (3)	2020	1.34	NA	Yes	dry tap	CD13, CD38, CD45, HLA-DR	NA	210	3	DA with Dasatinib	2

a, month; b, not detected; c, cytarabine; d, vincristine and prednisone; e, methotrexate; f, 6-mercaptopurine; g, Imatinib; h, daunorubicin and cytarabine; i, idarubicin and cytarabine; j, idarubicin, thioguanine and cytarabine; k, cord blood transplantation; l, homoharringtonine and azacitidine; m, adriamycin, cytarabine and etoposide; n, idarubicin, fludarabine and cytarabine; o, pirarubicin and cytarabine; p, mitoxantrone and etoposide; q, fludarabine and cytarabine; r, hydroxycamptothecin and etoposide.

**Table:** 

B. 9 cases of de novo Ph+ AMKL.											
Authors	Year	Peripheral		Splenomegaly	BM Characteristics					Treatment	Survival (mos^s^)
		WBCs (x10^9/L)	Basophils (%)		Blasts (%)	Immunophenotype	Cytogenetics	BCR/ABL	Fibrosis		
Cuneo A et al. (16)	1996	29	NA	Yes	58	CD7,CD33,CD41,TdT	46,XX, t(9;22)(q34;q11), t(2;9)(q12;q33)	NA	ND	NA	8
Mozziconacci MJ et al. (17)	1998	NA	NA	No	NA	NA	46,XX,inv(3)(q21q26)[4]/46,idem,t(9;22)(q3 4;q11)[15]	NA	NA	NA	Died
Balatzenko G et al. (18)	2004	1.1	NA	No	48	CD2,CD3,CD4,CD5,CD7,CD10,CD13,CD14,CD16,CD19,CD2 0,CD22,CD33,CD34,CD38,CD 56,CD61,CD62,HLA-DR	47,XX,+8,t(9; 22)(q34;q11)	P190	ND	NA	Died
Soupir CP et al. (19)	2007	23.2	0	Yes	93	ND	46,XY,t(9;22;21)(q34;q11;p11)[20]	P210	ND	NA	15
		15.3	2	No	28	ND	46,XY,t(9;22)(q34;q11)[20] 46,XY,t(9;22)(q34;q11)[1]/46XY,t(5;11) (q13;21),t(9;22)(q34;q11)[4]/46XY,der(4),t(1;4)(q12;p16),t(5;11)(q13;q21),t(9;22)(q34; q11)[8]/46XY,der(4),t(1;4)(q12;p16),t(5;11) (q13;q21),del(6)(p21),t(9;22)(q34;q11),17,+ mar1[4]	P210	ND	NA	13
Papageorgiou SG et al. (20)	2010	22	NA	Yes	50	CD34+,CD61+		P210	ND	IDA-FLAG^n^+IA +Dasatinib	24
Han ying et al. (21)	2014	3.63	NA	No	61.2	CD7,CD13,CD33,CD34,CD41, CD61,CD64,CD123,HLA-DR	46,XY,t(9;22)(q34;q11)[3]/46,XX[2]	NA	ND	TA^o^+ME^p^+FL AG^q^+HP^r^+IM	Died
Min GJ et al. (22)	2018	NA	NA	No	NA	NA	46,XX,t(9;22)(q34;q11.2)[20]	NA	ND	IA+IM	Died
Kashima E et al. (23)	2021	2.2	2	No	NA	CD13,CD34,CD117,HLA-DR	44,XX,der(3)(p21),add(4)(q21),add(5)(q22), del(15)(q11.2q15),-16add(17)(q25),-19	NA	2	IA	4

n, idarubicin; fludarabine and cytarabine; o, pirarubicin and cytarabine; p, mitoxantrone and etoposide; q, fludarabine and cytarabine; r, Hydroxycamptothecin and etoposide.

Key distinguishing features between CML-MKBC and Ph+ AMKL emerged from this analysis. Splenomegaly was present in almost all reported CML-MKBC cases but is rare in *de novo* Ph+ AMKL. Basophilia (≥2% in peripheral blood or BM) was present in 80% of CML-MKBC cases, whereas it was observed in only isolated cases of Ph+ AMKL and never exceeded 2%. Regarding BCR::ABL1 transcript types, the p210 isoform was uniformly detected in CML-MKBC, whereas p190 was also observed in *de novo* Ph+ AMKL. Notably, leukocytosis with elevated white blood cell counts was a characteristic feature of CML-MKBC, whereas no consistent changes were observed in Ph+ AMKL. BM fibrosis (grade II-IV) was present in both entities but tended to be more severe in CML-MKBC. Karyotype analysis showed that CML-MKBC had clonal evolution such as double Ph, while Ph+ AMKL was accompanied by different complex abnormalities. Pullarkat et al. (11) used targeted FISH technology to confirm that BCR::ABL1 fusion signal existed not only in primordial megakaryocytes, but also in differentiated neutrophils, and the signal may be enhanced in primordial cells, which was the decisive evidence for the diagnosis of CML-BP. Conversely, if the signal is limited to blast cells, it is more likely to be primary Ph+ AMKL (19). Because BCR::ABL1 expression in CML-BP is “systemic” and present throughout the myeloid lineage; In contrast, *De novo* Ph+ AMKL expression is “focal” and restricted to the leukemic cells themselves. Therefore, determining the breadth of BCR::ABL1 clones is also the key to differentiate CML-MKBCs from Ph+ AMKL. When we encounter suspected Ph+ AMKL patients in clinical, in addition to BM and laboratory examination, we must also inquire whether there are features such as splenomegaly or basophilia. If conditions permit, it is recommended to use targeted FISH and other techniques to search for evidence of BCR::ABL1 in differentiated myeloid cells for differentiation from CML-MKBC. Unfortunately, targeted FISH testing was not performed in this case due to the unavailability of this technology in our laboratory, therefore, definitive proof of clonal origin of the megakaryoblasts from the Ph+ stem cell could not be established. However, the absence of a prior CP history and the presence of pan-myeloid dysplasia make the diagnosis of Ph+ AMKL less likely. Based on the existing records at present, we have summarized the key points for differentiating CML-MKBC and Ph+ AMKL, as detailed in **Table 2**.

**Table 2:** Major points of differentiation between CML-MKBC and Ph+ AMKL.

Items	Favor to CML-MBC	Favor to Ph+ AMKL
Previous history	Acute onset, no prior CML history	
Clinical feature	Common splenomegaly and basophilia(>2%)	Rare splenomegaly and basophilia
BM blasts	20~60%	28~95% (often markedly higher)
Immunophenotype	CD41+/CD61/CD42	
Cytogenetics	100% Ph chromosome positive, additional clonal chromosomal, such as double Ph	del (3),der (4), del (6),del (15), +8, -19, +mar1, etc.
BCR-ABL type	P210	Predominantly P210, also P190
Fibrosis	2~4	Hardly ever

**Figure 6:**
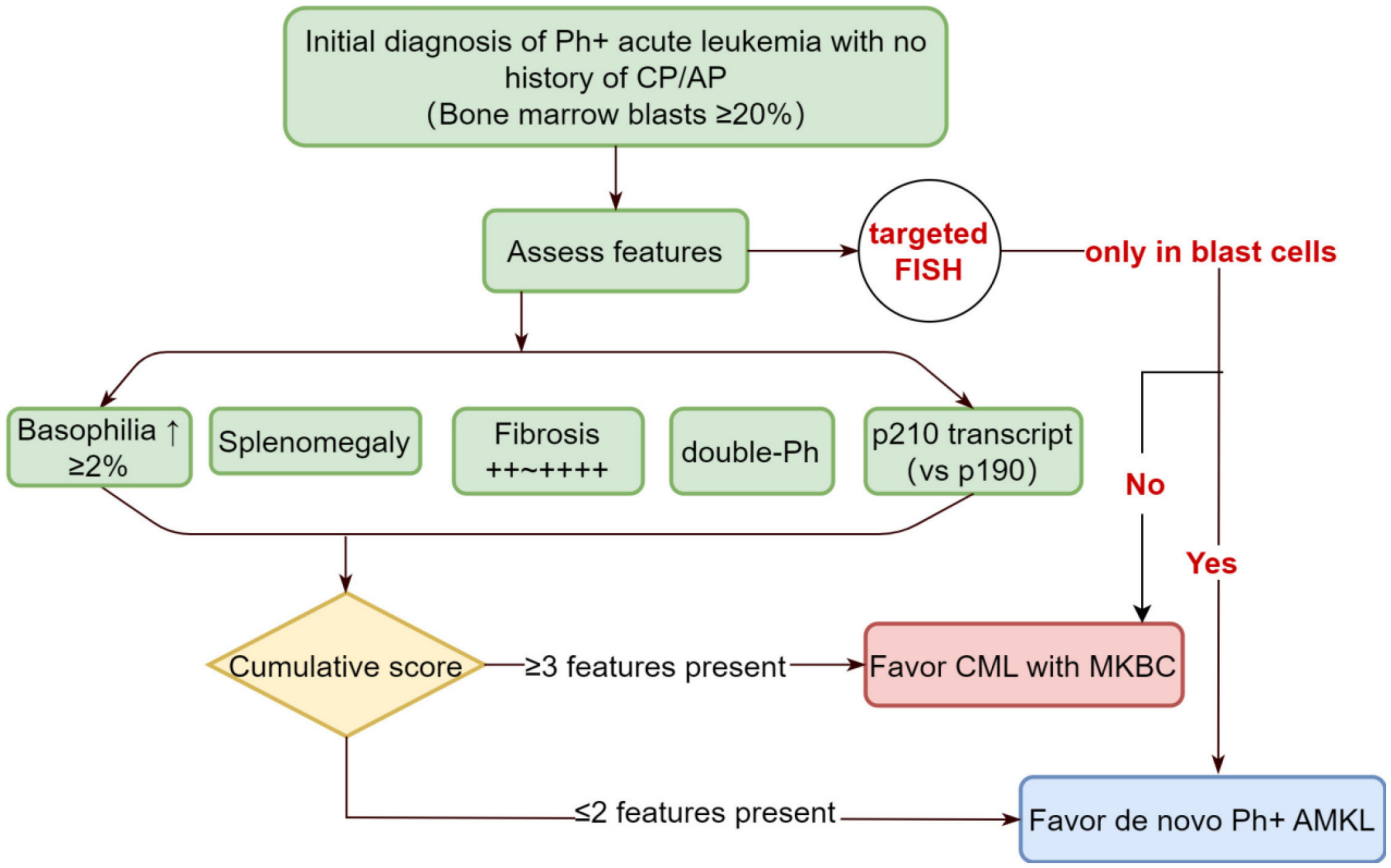
Literature search strategy for CML with megakaryocytic crisis and *de novo* Ph+ AMKL.

Distinguishing CML-MKBC from *de novo* Ph+ AMKL is challenging but clinically relevant. To address this diagnostic dilemma, we propose a stepwise diagnostic algorithm grounded in WHO 2022 (24) and ICC classifications (25) (**Figure 6**). Applying this algorithm to our case, upon presentation, BM showed 52% megakaryoblasts and had no history of CML-CP. The presence of four features—BCR::ABL1(p210) transcript was positive, 10.5% basophilia in BM, grade III fibrosis, and splenomegaly further evidence that CML may present initially in MKBC. Some studies have found that +Ph is more common in different stages of CML (26). And the karyotype showed that 15 cells harbored the Ph chromosome as the first clone, the original first clone acquired new genetic abnormalities during disease progression and evolved into a more malignant subclone, that is, two cells found to be tetraploid (92, XXYY) and contain double-Ph, which is a typical “clonal evolution” (27). It is also one of the gold standards for the diagnosis of CML-BP (28). In addition, ASXL1 and PTPN11 detected in genes are the common mutation spectrum of CML-BP, thereby driving cell clonal hematopoiesis and leading to the occurrence of hematological tumors (29–31). The quantification of high BCR::ABL1(p210) expression in this patient indicated that there were a large number of leukemia clones with active proliferation in the body. He may have had ASXL1 mutation in the early stage of the disease to accelerate clonal hematopoiesis, resulting in the megakaryoblast state in the BM at the time of the disease. Although the CSF1R gene has not clearly existed in the BP of CML, modeling study (32) has shown that hematopoietic stem cells expressing the CSF1R fusion gene can induce myeloproliferative tumor-like lesions and eventually progress to MKBC.

Although karyotyping revealed t (9;22) (q34; q11.2) as the sole abnormality, cryptic gene fusions cannot be entirely excluded. Standard karyotyping has limited sensitivity for submicroscopic rearrangements (33). Recent reports have described AML with concurrent BCR::ABL1 (p190) and RUNX1::MECOM fusions, associated with poor prognosis and TKI resistance (34, 35). While our patient’s p210 transcript, basophilia, splenomegaly, double Ph and high-grade myelofibrosis strongly support CML-MKBC, comprehensive RNA sequencing was not performed. Therefore, additional cryptic rearrangements cannot be definitively ruled out.

### Therapeutic strategy

There is no unified standard for the treatment of CML-MKBC, so there is a great challenge in the treatment. Less than 40% of CML patients in AP or BP achieved complete cytogenetic response (CCyR) using single-agent TKI or chemotherapy alone, and most patients relapsed within 6 months or 1 year (36–38). Therefore, the combination of the two may be an effective approach. Early studies have found that TKI can combine with chemotherapy drugs for the treatment of CML in blast crisis to produce synergistic effects (39, 40). According to NCCN and ELN guidelines (41, 42), TKI combined with chemotherapy is the first choice for induction therapy in CML-BP. The known median survival time is 3–30 months, and the best one is 27.4 months (TKI+ azacitidine) (43). Dragana et al. (44) found that chemotherapy drugs such as cytarabine show synergistic anti-leukemic activity when combined with TKIs, and the use of FLAG-IDA regimen combined with dasatinib can induce deep CR in CML-BP, and subsequent HSCT in patients can further improve survival. Nowadays, TKI have been iteratively developed and applied to various system diseases, and new drugs such as Dasatinib, Nilotinib and Ponatinib are common. Imatinib accounted for the largest proportion of TKI selection and performs well in the treatment of all stages of CML (7, 45). Moreover, the CSF1R mutant gene of this patient is one of the targets of imatinib, which can be used in AMKL patients expressing activated CSF1R (32). Fruehauf et al. (46) showed that the TKI+MA regimen showed excellent efficacy in combination with TKI, achieving an unprecedented high response rate (100%) in patients in CML-BP. High-dose imatinib combined with low-intensity chemotherapy also achieved a 92% CR in lymphoid blast crisis of CML (47).

Saikia et al. (48) found that lymphoid blast crisis of CML carries a 46.7% incidence of meningeal leukemia within 6 months, our patient had 52% megakaryocytic blasts, so similar considerations are warranted. In addition, imatinib penetrates the CNS poorly due to P-glycoprotein efflux, increasing the risk of metastasis in the central nervous system (CNS) (49, 50). For these consideration, five cycles of intrathecal methotrexate and cytarabine were administered as CNS prophylaxis. Our patient was treated with MA combined with imatinib as the first-line chemotherapy, followed by medium-and high-dose cytarabine and HA consolidation, long-term oral imatinib maintenance, and now has a survival time of 35 months. The first review of BM showed CR, BCR::ABL1^IS^(p210) significantly decreased also verified the effectiveness of imatinib combined with MA chemotherapy regimen, and further confirmed the favorable survival prognostic factor of imatinib. The BCR::ABL1^IS^(p210) level decreased from baseline 144.21% to 1.07% after the first combined chemotherapy, which also confirmed the existence of a high load of leukemic clone cells in the BM and the excellent effect of MA combined with imatinib (51). Finally, the negative conversion of fusion gene confirmed the necessity of subsequent consolidation therapy. We have achieved great result in this case, and the key lies in the choice of first-line treatment. In this particular case, the choice of first-line treatment (MA plus imatinib) appeared to contribute to the favorable outcome, suggesting that this combination may be considered as an option for similar patients. The concomitant chromosomal abnormalities and gene mutations may lead to TKI resistance in CML-MKBC, but MA chemotherapy bypasses this pathway, kills blasts rapidly and efficiently, and erases many proliferated but not resistant subclones. Imatinib precisely inhibited the BCR::ABL1(p210) signaling pathway and inhibited the proliferation of driver clones from the root (52). These two factors synergistically played the role of 1 + 1 > 2 to achieve deep MR5 (53), followed by long-term sustained inhibition of BCR::ABL1(p210) clones by TKI and accurate MRD monitoring to achieve long-term disease CR. Repeat testing of these mutations during CR was not performed, as the patient remained in sustained deep molecular response and clinical management did not necessitate repeat gene detection. Consequently, we cannot determine whether these mutations persisted at low levels or were eliminated with therapy. However, the achievement of sustained deep molecular response (MR5) suggests effective suppression of the BCR::ABL1(p210) driven clone.

Our patient refused HSCT due to financial difficulties, achieved hematologic CR and MR5 after the combination of TKI and chemotherapy, indicating that this combination regimen has the potential to induce MR, and survived for 35 months without HSCT, which exceeds the median survival reported in all current major case series. Tolerance of intensive therapy, and a CR to treatment may have contributed to this favorable outcome. However, we must emphasize that the long-term risk of recurrence in this patient remains very high. JNCCN (54) indicated that the median survival time of patients in CML-BP was only 6–11 months, and patients with double-Ph abnormalities were more likely to develop TKI resistance. It is possible for patients in CML- BP to achieve durable survival without HSCT, but this situation remains exceptional and HSCT should still remain the best chance of cure for patients in CML- BP (55).

### The fast track of CML

This study reports an extremely rare case of CML with MKBC as the initial presentation. This aggressive presentation raises the question of why disease progression can sometimes “skip” the CP. Combining the molecular features of this case with the existing literature, we proposed a reasonable hypothesis that a BCR::ABL1 clone harboring high-risk cooperative mutations early in development may directly drive a “fast-track” lineage-specific transformation—toward MKBC—within a profibrotic BM microenvironment. Based on the molecular features of this case and the existing literature, we offer the following hypothesis-generating interpretation: a BCR::ABL1 clone harboring high-risk cooperative mutations early in development may potentially drive a “fast-track” lineage-specific transformation—toward MKBC—within a profibrotic BM microenvironment. We acknowledge that this interpretation is speculative and derived from a single case; it is intended to generate hypotheses for future studies rather than to establish definitive pathogenic pathways.

First, Complex clonal evolution karyotype and high level of BCR::ABL1 quantification were detected in the patient at the time of initial diagnosis, suggesting that the BCR::ABL1 clone has inherent high genomic instability, and additional genetic epigenetic damage cooperation is the basis of rapid blast transformation (56). More importantly, ASXL1 and PTPN11, two known BP driver mutations in CML, were detected by next-generation sequencing. Although the classical RUNX1 mutation, which blocks megakaryoblastic differentiation, was not detected in this case (57, 58), the epigenetic disorder caused by ASXL1 mutation may contribute to the aberrant activation of certain lineage-specific genes, though the extent of this effect likely varies across different genetic contexts. As an epigenetic regulator (59), ASXL1 is present in both CP and BP of CML (60), and its inactivation has been associated with widespread alterations in hematopoietic differentiation programs, but the precise mechanisms underlying this reprogramming and their relevance to human CML-BP remain incompletely defined. PTPN11 (61, 62) is a potent activating mutation of the RAS/MAPK signaling pathway, which in concert with BCR::ABL1, may confer explosive proliferation ability to skip the CP and accelerate into the BP of leukemic clones. Secondly, under high concentration of TGF-β microenvironment (63), this high-risk clone may be abnormally “directed” to megakaryocyte differentiation, and at the same time form a large accumulation of primitive megakaryocytes due to the block of differentiation and maturation. The presence of basophils in the patient’s BM, up to 10.5%, was a residual marker of the presence of the CML pluripotent stem cell clone, supporting that the BP occurred on top of an established CML background. Finally, this case had severe myelofibrosis (grade III) at diagnosis, which was not a passive consequence of BP but may have been an active participant in pathogenesis. TGF-βand other factors secreted by BCR::ABL1 positive cells drive fibrosis, and the fibrotic network with its accompanying inflammatory environment can in turn support the survival, proliferation and abnormal differentiation of leukemia stem cells through abnormal biomechanical signals and cytokine networks. Severe fibrosis is strongly associated with an increased risk of CML entering the AP or BP (64). This self-reinforcing vicious cycle may provide the necessary soil for the “leapfrog” progression of high-risk clones.

### Limitations

Several limitations should be acknowledged. First, as a single case report, our findings may not be generalizable, and the proposed diagnostic and therapeutic framework requires validation in larger cohorts. Second, targeted FISH to determine the lineage distribution of BCR::ABL1—the gold standard for distinguishing CML-BP from *de novo* Ph+ AMKL—was not available in our laboratory, leaving some diagnostic uncertainty. Third, repeat mutation testing for ASXL1, PTPN11, and other detected mutations during complete remission was not performed, as the patient achieved sustained deep MR and clinical management did not necessitate re-evaluation. Fourth, the patient declined HSCT due to financial constraints, limiting our ability to assess the potential benefit of this curative approach. Finally, the proposed “fast-track” pathogenic hypothesis is speculative and requires independent validation.

## Conclusion

**Figure 7:**
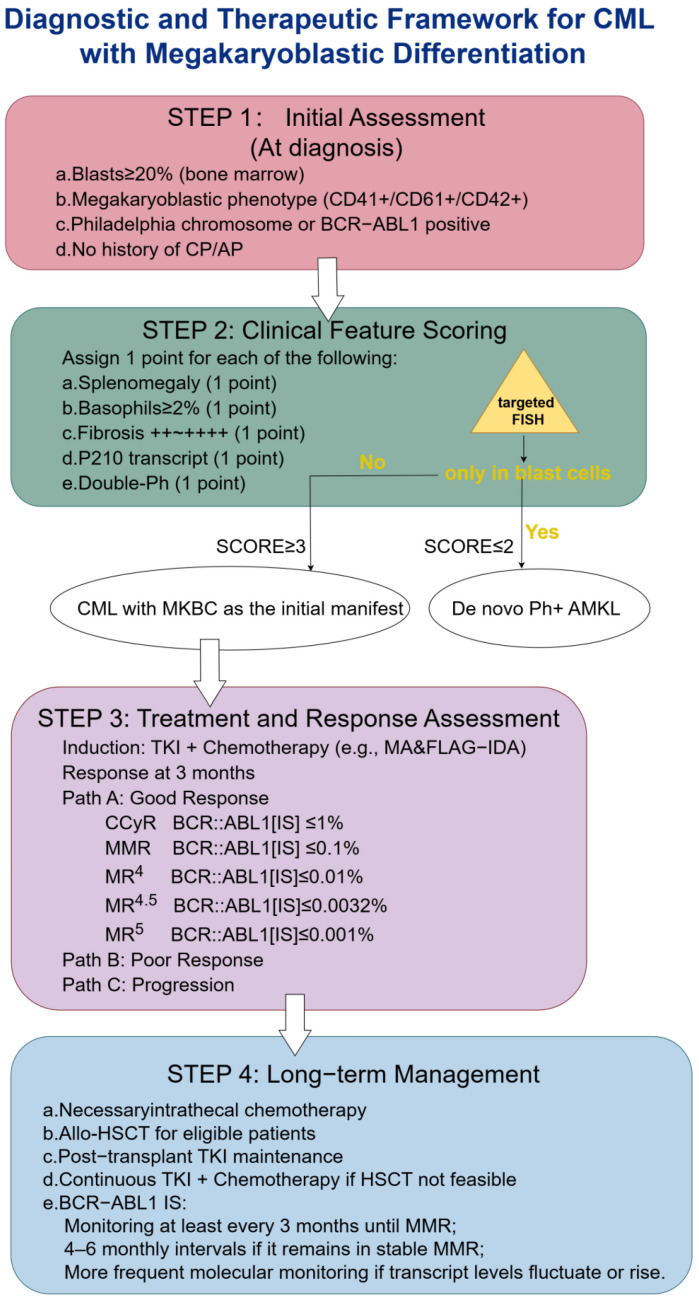
Diagnostic and therapeutic framework to distinguish CML with megakaryocytic differentiation from *de novo* Ph+ AMKL.

CML-MKBC is a rare entity associated with a poor prognosis. The following features may aid in distinguishing CML-MKBC from Ph+ AMKL: splenomegaly, basophilia (≥2%), double Ph, high-grade myelofibrosis, and BCR::ABL1(p210) positivity. However, definitive distinction ideally requires targeted FISH to assess BCR::ABL1 distribution across myeloid lineages. If targeted FISH is used, the diagnosis will be more definite. Imatinib with MA/HA and cytarabine-based chemotherapy have proven beneficial in improving the disease outcome to the extent of achieving MR5 as in our patient. HSCT stills remain the preferred option for achieving a high cure rate and is recommended for patients in CML- CP as early as possible (65). Of course, we acknowledge that lineage-specific FISH studies were not performed, leaving some diagnostic uncertainty. Nevertheless, our case suggests that CML may occasionally present initially in MKBC and suggests an integrated diagnostic treatment framework (**Figure 7**).

## Data Availability

The original contributions presented in the study are included in the article/supplementary material. Further inquiries can be directed to the corresponding author/s.
